# Relationship of neighborhood social determinants of health on racial/ethnic mortality disparities in US veterans—Mediation and moderating effects

**DOI:** 10.1111/1475-6773.13547

**Published:** 2020-08-29

**Authors:** Michelle S. Wong, W. Neil Steers, Katherine J. Hoggatt, Boback Ziaeian, Donna L. Washington

**Affiliations:** ^1^ VA HSR&D Center for the Study of Healthcare Innovation, Implementation & Policy (CSHIIP) VA Greater Los Angeles Healthcare System Los Angeles CA USA; ^2^ San Francisco VA Healthcare System San Francisco CA USA; ^3^ Department of Medicine University of California San Francisco CA USA; ^4^ Division of Cardiology, Department of Medicine David Geffen School of Medicine at UCLA Los Angeles CA USA; ^5^ Division of General Internal Medicine and Health Services Research, Department of Medicine David Geffen School of Medicine at UCLA Los Angeles CA USA

**Keywords:** neighborhood deprivation, Racial/ethnic disparities, residential segregation, social determinants of health, Veterans

## Abstract

**Objective:**

To examine mediation and moderation of racial/ethnic all‐cause mortality disparities among Veteran Health Administration (VHA)‐users by neighborhood deprivation and residential segregation.

**Data sources:**

Electronic medical records for 10/2008‐9/2009 VHA‐users linked to National Death Index, 2000 Area Deprivation Index, and 2006‐2009 US Census.

**Study design:**

Racial/ethnic groups included American Indian/Alaskan Native (AI/AN), Asian, non‐Hispanic black, Hispanic, Native Hawaiian/Other Pacific Islander, and non‐Hispanic white (reference). We measured neighborhood deprivation by Area Deprivation Index, calculated segregation for non‐Hispanic black, Hispanic, and AI/AN using the Isolation Index, evaluated mediation using inverse odds‐weighted Cox regression models and moderation using Cox regression models testing for neighborhood*race/ethnicity interactions.

**Principal findings:**

Mortality disparities existed for AI/ANs (HR = 1.07, 95%CI:1.01‐1.10) but no other groups after covariate adjustment. Neighborhood deprivation and Hispanic segregation neither mediated nor moderated AI/AN disparities. Non‐Hispanic black segregation both mediated and moderated AI/AN disparities. The AI/AN vs. non‐Hispanic white disparity was attenuated for AI/ANs living in neighborhoods with greater non‐Hispanic black segregation (*P* = .047). Black segregation's mediating effect was limited to VHA‐users living in counties with low black segregation. AI/AN segregation also mediated AI/AN mortality disparities in counties that included or were near AI/AN reservations.

**Conclusions:**

Neighborhood characteristics, particularly black and AI/AN residential segregation, may contribute to AI/AN mortality disparities among VHA‐users, particularly in communities that were rural, had greater black segregation, or were located on or near AI/AN reservations. This suggests the importance of neighborhood social determinants of health on racial/ethnic mortality disparities. Living near reservations may allow AI/AN VHA‐users to maintain cultural and tribal ties, while also providing them with access to economic and other resources. Future research should explore the experiences of AI/ANs living in black communities and underlying mechanisms to identify targets for intervention.

**What this study adds**

- Even within health care systems that strive to provide equal access to care to all patients, such as the Veterans Health Administration, racial/ethnic disparities in mortality exist
- Research in the general population suggests that neighborhood deprivation and segregation contribute to racial/ethnic disparities, but it is unknown whether these neighborhood characteristics also account for racial/ethnic disparities among Veteran Health Administration (VHA) users.
- This study identified all‐cause mortality disparities among American Indian/Alaskan Native (AI/AN) VHA‐users versus non‐Hispanic whites and that non‐Hispanic black and AI/AN neighborhood segregation both mediated these disparities.
- The disparity in all‐cause mortality among AI/AN VHA‐users was attenuated in neighborhoods with greater non‐Hispanic black segregation, suggesting the importance of neighborhood‐level social determinants of health on racial/ethnic mortality disparities.

## 1 INTRODUCTION

Health care is an important—but not sole—determinant of health outcomes.^1^ Even within health care systems that strive to provide equal access to all patients, racial/ethnic disparities exist.^2^, ^3^, ^4^, ^5^, ^6^ Health care systems alone cannot expect to eliminate racial/ethnic health disparities. Eliminating disparities will require examining social determinants of health: social and contextual factors outside of health care systems that affect health.

The Veterans Health Administration (VHA) is an example of a health system that has made strides in addressing racial/ethnic disparities. Within VHA, non‐Hispanic blacks (hereafter, “blacks”) had similar or lower mortality from prostate cancer, lung cancer, and chronic kidney disease relative to non‐Hispanic whites (hereafter, “whites”).^2^, ^7^, ^8^ However, some VHA racial/ethnic disparities still persist, including heart disease mortality among blacks, all‐cause mortality among American Indian/Alaskan Natives (AI/ANs), and hypertension control in both of these groups.^2^, ^4^, ^5^

The neighborhoods in which people live are an important social determinant of health. Methodological advances have allowed researchers to disentangle the neighborhood's compositional effects (ie, individuals with worse health cluster in certain neighborhoods, for example, sicker people live in lower income communities) from its contextual effects on health (ie, group properties of the neighborhood influence health, for example, neighborhood poverty leads to worse health).^9^ Studies in the US general population have identified neighborhood contextual effects, reinforcing that where we live does, indeed, affect our health.^10^

While neighborhoods encompass complex social and physical environments, identifying specific neighborhood characteristics associated with health disparities may guide policy and practice, such as community‐based partnerships, initiatives to improve healthy food access, and comprehensive neighborhood revitalization efforts.^11^, ^12^, ^13^, ^14^ Two neighborhood characteristics associated with health disparities in the general population are neighborhood socioeconomic status (SES) and racial/ethnic segregation.^10^, ^15^, ^16^, ^17^ Lower SES neighborhoods typically have fewer health promoting resources (eg, healthy food options, recreational spaces), more crime, and subpar housing, which negatively affect health. Furthermore, lower SES and racial/ethnic minority communities have fewer health care resources.^18^, ^19^

Racial/ethnic disparities may arise from racial/ethnic groups living in different neighborhoods that expose them to different contexts and risk factors. In the US general population, racial/ethnic minority groups at higher risk for mortality, such as blacks, are more likely to live in segregated and more deprived neighborhoods.^20^ However, segregation can also have positive health effects for racial/ethnic minority groups by buffering residents from racism and other stressors, attracting minority‐owned businesses that provide culturally concordant services (eg, traditional food outlets), providing spaces like churches for groups to congregate and organize, and fostering social connections that promote transmission of health‐related information (eg, awareness of health care service availability).^10^, ^21^, ^22^

Segregation effects on health may vary by race/ethnicity. Segregation of different racial/ethnic groups arose through different processes and historical forces. Black segregation formed through discriminatory practices and policies (eg, residential redlining concentrated blacks into low SES, disinvested communities).^20^ AI/AN segregation occurred through European and American colonization that led to forced and traumatic relocation of AI/ANs onto tribal reservations.^23^ Many reservations are located in rural areas with high poverty and unemployment and limited health care resources .^23^, ^24^ However, residing on reservations may also promote health by strengthening tribal and cultural ties.^25^ Hispanic segregation has occurred through the creation of ethnic enclaves. Despite being located in disproportionately low SES areas,^26^ Hispanic enclaves may promote health by preserving cultural practices and promoting social cohesion.^27^

It is unknown whether neighborhood deprivation and segregation account for observed disparities among racial/ethnic minority VHA‐users. Understanding whether such relationships exist in this population can yield important insights for the VHA and the broader health care system. A key difference between VHA‐users and the US general population is that VHA‐users have access to a health care system that provides comprehensive physical and mental health services and has initiatives to improve access (eg, transportation and extended clinic hours).^28^ Given that unequal access to health care contributes to racial/ethnic disparities,^29^ examining drivers of racial/ethnic disparities among VHA‐users provides a unique opportunity to understand how social determinants contribute to disparities beyond health care access.^30^ VHA‐users, compared to the US general population, have lower incomes and poorer health status;^31^, ^32^ therefore, with our analysis, we can examine neighborhood influences within a population with medical and social vulnerabilities.

This study has specific implications to VHA, given its broad mission to improve the health of all Veterans. Compared to other health care systems, VHA may be better positioned to address social determinants of health, as they are already targeting some determinants including housing and employment. For example, VHA’s permanent supportive program is one of the largest programs in the nation and has been credited with substantially reducing Veteran homelessness nationally.^33^ VHA also has employment training programs for those with service‐connected disability and serious mental illness.^34^ It is unknown, though, whether these efforts to improve Veteran health and well‐being buffer Veterans from some of the deleterious effects of neighborhood‐level social determinants of health.

In this study, we examined the role of two neighborhood‐level social determinants of health on racial/ethnic all‐cause mortality disparities among a cohort of Veteran VHA‐users—neighborhood deprivation and residential segregation. Our first aim explored racial/ethnic differences in all‐cause mortality from 2008 to 2016 in a national cohort of VHA‐users. Our second aim examined whether neighborhood deprivation a) mediated or b) moderated racial/ethnic all‐cause mortality disparities among VHA‐users. Our third aim similarly examined whether residential segregation a) mediated or b) moderated these relationships. We hypothesized that blacks and AI/ANs would experience disparities in all‐cause mortality, that both neighborhood deprivation and segregation would mediate the racial/ethnic all‐cause mortality disparities, and that racial/ethnic disparities would be greater in neighborhoods with greater deprivation and segregation and attenuated in less deprived and segregated neighborhoods.^23^

## 2 METHODS

### 2.1 Data and sample

Our analytic sample consisted of a national cohort of Veterans (age ≥ 18 years) with VHA ambulatory care use from 10/2008 to 9/2009 (fiscal year [FY] 2009). Data came from VHA’s electronic medical records, linked to the Centers for Disease Control and Prevention (CDC)’s National Death Index (NDI) to provide mortality data, the 2000 Area Deprivation Index (ADI)^35^ to provide neighborhood SES information, and the 2006‐2009 US Census to provide residential segregation information. Patient records were linked to the NDI based on exact matches of social security numbers and dates of birth, to the ADI by census block group identifiers, and to the US Census by census tract identifiers. We excluded individuals with duplicate NDI records (*n* = 13), invalid dates of index ambulatory care visits (*n* = 4,778), and missing data on covariates (due to missing or mismatched census tract identifiers, models with ADI excluded *n* = 572,882 [11.4 percent of sample], models with segregation excluded *n* = 577,700 [11.5 percent of sample]). Mortality was ascertained through 12/2016.

### 2.2 Measures

#### 2.2.1 Dependent variables

We examined time to all‐cause mortality, calculated as the difference in years between date‐of‐death and qualifying date‐of‐initial FY2009 health care use. Deaths were classified as either observed on the date of death or administratively censored at the end of the mortality ascertainment period.

#### 2.2.2 Independent variable

Our main independent variable was a categorical indicator of patient race/ethnicity. We created the race/ethnicity variable using a validated algorithm that combines multiple data sources and years of data in a hierarchy ordered by data quality of the source data files, with self‐identified race/ethnicity considered the highest.^36^, ^37^ We classified all individuals with Hispanic ethnicity as Hispanic and all others by race. Our analysis included the following racial/ethnic groups: non‐Hispanic AI/AN (hereafter, “AI/AN”), non‐Hispanic Asian (hereafter, “Asian”), black, Hispanic, non‐Hispanic Native Hawaiian/Other Pacific Islander (hereafter, “NH/OPI”), and white, as well as multirace and unknown race (results not presented for latter two groups).

#### 2.2.3 Mediators and moderators

We examined two neighborhood characteristics: neighborhood deprivation and neighborhood segregation. Neighborhood deprivation was captured through the ADI.^35^ This validated index was derived from 17 measures of income, education, employment, and housing quality from the 2000 American Community Survey (ACS) data.^38^, ^39^ Higher ADI values indicate greater neighborhood deprivation.

While numerous measures of neighborhood segregation have been developed to measure residential segregation,^40^ our analysis used the isolation index, which captures the degree to which a member of one racial/ethnic group is exposed only to individuals of that same group.^40^ The isolation index has several advantages over other segregation measures for our analysis: it better captures the underlying processes through which segregation can negatively affect health (eg, concentrated disadvantage) and is one of the most commonly used segregation indices.^17^, ^41^ The isolation index is calculated by: $\sum_{i=1}^{n}\left[\left(x_{i}/X\right)\left(x_{i}/t_{i}\right)\right]$, where n is the number of census tracts in the county, x_i_ is the comparison group population in census tract i, X is the sum of all x_i_ (total comparison group population in the county), and t_i_ is the total population of census tract i. The isolation index ranges from 0 to 1, where higher isolation indices indicate greater racial/ethnic group isolation. We calculated separate isolation indices for blacks, Hispanics, and AI/ANs.

#### 2.2.4 Other variables

We included the following covariates in our analysis: age (categorical: 18‐29, 30‐39, 40‐49, 50‐59, 60‐69, 70‐79, and 80+), sex, medical comorbidity, mental health comorbidity [categorical: serious mental illness (SMI; defined as a diagnosis of schizophrenia, schizoaffective or bipolar disorder, or other psychoses), depression without SMI, other mental health diagnosis without SMI or depression, and no mental health diagnosis], individual‐level SES (high SES, low SES, indeterminant SES), and rurality indicator (urban, rural, highly rural). The medical comorbidity index was adapted from the Seattle Index of Comorbidity, based on a weighted count of smoking status and seven chronic medical conditions associated with increased mortality (prior myocardial infarction, cancer, lung disease, congestive heart failure, diabetes, pneumonia, and stroke).^42^ We identified medical and mental health diagnoses using the ICD‐9‐CM outpatient and inpatient diagnosis codes from FY2009. Individual‐level SES was based on VHA’s enrollment priority group. Veterans that did not have a service‐connected disability, but that had an income below VHA’s threshold for requiring copayment for care were classified as having low SES, while those above the threshold were classified as high SES. For Veterans with a military service‐connected disability, their priority group did not reflect their income; thus, they are classified as indeterminate SES. Rurality indicator categories were based upon the Goldsmith Modification of the Office of Management and Budget definition of urban, rural, and highly rural land areas.^43^

### 2.3 Statistical analysis

We calculated descriptive statistics of race/ethnicity stratified means and proportions of the VHA population's demographics, health characteristics, and neighborhood characteristics, and crude race/ethnicity‐stratified mortality rates (number of deaths/100 000 population).

#### 2.3.1 Racial/ethnic all‐cause mortality differences

We used three Cox regression models to calculate all‐cause mortality hazard ratios comparing each racial/ethnic minority group to whites (reference group). Model 1 adjusted for age and sex. Model 2 further adjusted for individual SES and urban/rural status, which are important factors when considering neighborhood‐level social determinants of health. Model 3 further adjusted for comorbidity and mental health comorbidity, which are on the causal pathway of mortality disparities. All models included clustered standard errors at the VHA facility level to account for within‐VHA facility correlation.

#### 2.3.2 Mediation analysis

Mediation analysis involves estimating the total effect of race/ethnicity on mortality, and decomposing this effect into its direct (unmediated) and indirect (mediated) effect components. We tested mediation by neighborhood deprivation and segregation separately in the following way. First, we estimated the total effect of race/ethnicity on mortality for each mediator through Cox regression while adjusting for potential confounders and the neighborhood mediator of interest. For each total effects model showing evidence of a racial/ethnic disparity relative to whites at *P* < .05, we subsequently estimated the direct and indirect effects of race/ethnicity with Tchetgen Tchetgen's inverse odds weighting approach (IOW)^44^ as described by Nguyen 2015’s practical guidance.^45^ IOWs were derived by fitting separate polytomous regression models estimating the relationship between race/ethnicity and each neighborhood characteristic mediators with covariate adjustment, and using the resulting coefficients to calculate an IOW for each observation in the nonwhite racial/ethnic groups (“treatment” groups), while each whites observation was given an IOW of 1 (“control” group). We then fitted inverse odds‐weighted Cox regression models to estimate direct effects of race/ethnicity on mortality while controlling for the same demographics and comorbidities from the total effects model. Next, we calculated the indirect effects of race/ethnicity on mortality by subtracting the direct effect coefficient from the total effect coefficient for racial/ethnic group disparities of interest, and bootstrapped estimates to obtain standard errors and 95% confidence intervals. We considered indirect effects statistically significant at *P* < .05 as evidence of mediation. Finally, we transformed the total, direct and indirect effect coefficients into hazard ratios and transformed the indirect effect coefficients into proportions of the total effect explained by the neighborhood characteristic mediator of interest (indirect effect/total effect).

#### 2.3.3 Moderation analysis

We examined whether neighborhood deprivation or neighborhood segregation modified racial/ethnic mortality differences. We modeled the neighborhood‐level modifiers in separate Cox regression models that included a product term between the moderator of interest and race/ethnicity, while controlling for all covariates described above in model 3’s specifications. If the interaction term was statistically significant at *P* < .05, we considered the neighborhood characteristic to be a mediator.

#### 2.3.4 Sensitivity analyses

We conducted two sensitivity analyses. First, urban‐rural differences may be important to consider because AI/ANs are more likely to reside in rural and highly rural communities, and AI/ANs residing in urban versus rural communities may differ in ways that influence mortality. Thus, for neighborhood characteristics that statistically significantly mediated or moderated AI/AN disparities, we also stratified analyses by urban vs. rural (combining rural and highly rural, due to small highly rural sample size). Second, since small population sizes can affect the stability of the segregation indices,^46^ and some counties have no or few AI/ANs, we examined mediating and moderating effects of AI/AN segregation in a subsample residing in Indian Health Service (IHS)’s Contract Health Service Delivery Area (CHSDA), which are counties that either include or adjacent to reservations.^24^

## 3 RESULTS

Within the cohort of 5,032,009 Veterans who were observed for 34,859,260 person‐years, there were 1,374,356 deaths. Cohort characteristics stratified by Veteran race/ethnicity are presented in Table 1. There were significant differences across racial/ethnic groups in all individual‐ and neighborhood‐level covariates (*P* < .05 for all variables). Whites were the oldest in the sample (mean age: 65.07 years, SD: 15.22). Blacks had the largest proportion of female Veterans (9.98 percent). Asians had the lowest medical comorbidity (mean: 1.26; SD: 1.77). AI/ANs had the highest levels of depression without SMI (23.41 percent) and other mental disorders without SMI or depression (13.52 percent). Whites were most likely to have high individual SES (28.79 percent). AI/ANs were most likely to reside in highly rural areas (10.43 percent). Neighborhood deprivation was lowest among Asians (87.48). Blacks and Hispanics were more likely to live in higher black‐segregated (mean: 0.45 SD: 0.22) and Hispanic‐segregated neighborhoods (mean: 0.52, SD: 0.29), respectively. Crude mortality proportions were highest among whites (4509 per 100 000).

**TABLE 1:** Veteran health administration user sample characteristics

	White(n = 3,767,310)	AI/AN (n = 23,337)	Asian (n = 33,895)	Black (n = 718,953)	Hispanic (n = 242,402)	NH/OPI (n = 29,276)	*P*‐value
Demographic characteristics							
Age, mean (SD)	65.07 (15.22)	57.57 (15.15)	56.33 (19.04)	56.47 (13.97)	56.98 (17.40)	60.35 (15.85)	<.01
Female, %	4.44	8.33	7.42	9.98	5.94	7.55	<.01
Medical comorbidity, mean (SD)^1^	1.93 (2.18)	1.94 (2.25)	1.26 (1.77)	1.89 (2.22)	1.60 (2.01)	1.91 (2.17)	<.01
Mental health comorbidity							
SMI^2^	4.26	5.47	4.27	7.47	6.48	5.79	<.01
Depression without SMI	18.30	23.41	13.34	18.29	21.02	20.36	
Other mental health disorders without SMI or depression	6.72	13.52	7.11	11.76	9.93	10.38	
No mental health diagnosis	70.71	57.60	75.28	62.48	62.56	63.47	
Individual SES							
High SES	28.79	12.03	14.28	13.15	11.70	18.22	<.01
Low SES	26.38	30.59	18.30	33.51	33.94	22.31	
Indeterminate	44.84	57.38	67.42	53.33	54.36	59.47	
Rurality indicator							
Urban	51.24	40.96	81.51	77.71	78.41	63.97	<.01
Rural	46.94	48.61	18.09	22.21	20.02	34.99	
Highly Rural	1.82	10.43	0.40	0.08	1.56	1.04	
Neighborhood characteristics							
Neighborhood deprivation^3^, mean (SD)	101.26 (15.07)	104.57 (12.65)	87.48 (20.89)	106.56 (12.37)	103.73 (14.59)	99.26 (16.85)	<.01
Black segregation^4^, mean	0.21 (0.19)	0.18 (0.18)	0.20 (0.16)	0.45 (0.22)	0.19 (0.19)	0.23 (0.20)	<.01
Hispanic segregation^4^, mean	0.18 (0.18)	0.20 (0.19)	0.33 (0.20)	0.23 (0.19)	0.52 (0.29)	0.25 (0.22)	<.01
AI/AN segregation^4^, mean	0.03 (0.06)	0.08 (0.14)	0.02 (0.04)	0.02 (0.04)	0.04 (0.09)	0.02 (0.05)	<.01
Crude mortality proportions per 100,000^5^							
All‐cause mortality	4509	3221	1959	2662	2524	3421	‐

Notes

AI/AN denotes American Indian/Alaska Natives, NH/OPI denotes Native Hawaiians/Other Pacific Islanders. 1. Medical comorbidity index on a scale of 0 (no medical comorbidities) to 15 (high comorbidity); 2. serious mental illness (SMI) defined as having a diagnosis of schizophrenia, schizoaffective disorder, bipolar disorder, or other psychoses; 3. neighborhood deprivation was measured by the Area Deprivation Index at the census block group level, where higher scores denote greater deprivation; 4. neighborhood segregation was measured by the Isolation Index at the census tract level, where higher values indicate greater isolation for blacks, Hispanics, and AI/ANs, respectively; 5. crude mortality proportions calculated as number of deaths/population *100,000; we did not calculate a test statistic for racial/ethnic group differences

### 3.1 Racial/ethnic all‐cause mortality differences

After adjusting for age and sex, AI/ANs (HR = 1.14, 95%CI: 1.10‐1.19) and blacks (1.02, 95%CI: 1.00‐1.04) had higher mortality versus whites (Table 2 and Table S1). With further individual SES and rurality adjustment, blacks had lower mortality (HR = 0.96, 95%CI: 0.94‐0.97), while the AI/AN disparity persisted (HR = 1.10, 95%CI: 1.05‐1.14). With additional medical and mental health comorbidity adjustment, these differences remained.

**TABLE 2:** Adjusted racial/ethnic all‐cause mortality differences relative to whites

	Model 1 Hazard ratio (95% CI)	Model 2 Hazard ratio (95% CI)	Model 3 Hazard ratio (95% CI)
Race/ethnicity			
White	Ref	Ref	Ref
AI/AN	**1.14 (1.10‐1.19)**	**1.10 (1.05, 1.14)**	**1.07 (1.03‐1.11)**
Asian	**0.63 (0.58‐0.68)**	**0.61 (0.55, 0.68)**	**0.67 (0.60‐0.74)**
Black	**1.02 (1.00‐1.04)**	**0.96 (0.94, 0.97)**	**0.93 (0.91‐0.94)**
Hispanic	**0.81 (0.76‐0.86)**	**0.74 (0.68, 0.81)**	**0.75 (0.68‐0.82)**
NH/OPI	0.98 (0.92, 1.04)	0.97 (0.93, 1.02)	**0.95 (0.91‐0.99)**

Notes

CI is confidence interval. Bold text denotes statistically significant association at *P* < .05. Model 1 adjusted for age and sex, Model 2 adjusted for age, sex, individual SES, and rurality. Model 3 adjusted for age, sex, Individual SES, rurality, and medical and mental health comorbidities.

Age‐ and sex‐adjusted mortality was lower in Asians (HR = 0.63, 95%CI: 0.58‐0.68) and Hispanics (HR = 0.81, 95%CI: 0.76‐0.86) and similar in NH/OPIs (HR = 0.98, 95%CI: 0.92‐1.04) versus whites. Differences for Asians and Hispanic remained after individual SES, rurality, and medical and mental health comorbidity adjustment. With additional medical and mental health comorbidity adjustment, NH/OPIs had lower mortality compared to whites (HR = 0.95, 95%CI: 0.91‐0.99).

### 3.2 Mediation

Analysis of whether neighborhood characteristics mediated the relationship between AI/AN race/ethnicity and mortality revealed that black segregation mediated AI/AN mortality disparities (indirect effect HR = 1.02, 95%CI: 1.00‐1.04, *P*‐value = .03), reducing the total effect of the AI/AN mortality disparity by 35.4% (Table 3). AI/AN segregation also mediated AI/AN disparities (indirect effect HR = 1.02, 95%CI: 1.00‐1.05, *P*‐value = .05). There was no evidence of medication by neighborhood deprivation (indirect effect HR = 1.02, 95%CI: 0.99‐1.04) or Hispanic segregation (indirect effect HR = 1.01, 95%CI: 0.99‐1.03).

**TABLE 3:** IOW Mediation of racial/ethnic differences in mortality among VHA‐users by neighborhood characteristics: total, direct, and indirect effects

Mediators	Total effect		Direct effect		Indirect effect		% change
	HR (95% CI)	*P*‐value	HR (95% CI)	*P*‐value	HR (95% CI)	*P*‐value	
AI/AN vs. white all‐cause mortality difference							
Neighborhood Deprivation	**1.06 (1.02 ‐ 1.10)**	**<.01**	**1.04 (1.00 ‐ 1.08)**	**.04**	1.02 (0.99 −1.04)	.18	28.98%
Black segregation	**1.07 (1.04 ‐ 1.11)**	**<.01**	**1.05 (1.01 ‐ 1.08)**	**.02**	**1.02 (1.00 ‐ 1.05)**	**.03** **	**35.40%**
Hispanic segregation	**1.07 (1.03 ‐ 1.10)**	**<.01**	**1.05 (1.01 ‐ 1.09)**	**.01**	1.01 (0.99 ‐ 1.03)	.15	21.70%
AI/AN segregation	**1.07 (1.03, 1.10)**	**<.01**	**1.05 (1.01, 1.08)**	**.02**	1.02 (1.00, 1.04)	.05*	32.89%
Asian vs. white all‐cause mortality difference							
Neighborhood Deprivation	**0.68 (0.66, 0.70)**	**<.01**	**0.75 (0.72, 0.78)**	**<.01**	**0.90 (0.88, 0.93)**	**<.01** **	**25.93%**
Black segregation	**0.65 (0.63, 0.68)**	**<.01**	**0.73 (0.70, 0.76)**	**<.01**	**0.89 (0.87, 0.92)**	**<.01** **	**26.53%**
Hispanic segregation	**0.65 (0.63, 0.67)**	**<.01**	**0.76 (0.73, 0.79)**	**<.01**	**0.86 (0.83, 0.88)**	**<.01** **	**36.28%**
AI/AN segregation	**0.65 (0.63, 0.67)**	**<.01**	**0.74 (0.71, 0.77)**	**<.01**	**0.88 (0.86, 0.91)**	**<.01** **	**29.02%**
Black vs. white all‐cause mortality difference							
Neighborhood Deprivation	**0.91 (0.90, 0.91)**	**<.01**	**0.93 (0.90, 0.95)**	**<.01**	0.98 (0.96, 1.01)	.13	20.14%
Black segregation	**0.91 (0.90, 0.91)**	**<.01**	**0.93 (0.92, 0.94)**	**<.01**	**0.98 (0.97, 0.99)**	**<.01** **	**21.31%**
Hispanic segregation	**0.93 (0.93, 0.94)**	**<.01**	**0.94 (0.93, 0.95)**	**<.01**	0.99 (0.99, 1.00)	.13	8.35%
AI/AN segregation	**0.93 (0.92, 0.94)**	**<.01**	**0.94 (0.92, 0.95)**	**<.01**	0.99 (0.98, 1.00)	.16	14.52%
Hispanic vs. white all‐cause mortality difference							
Neighborhood Deprivation	**0.74 (0.74, 0.75)**	**<.01**	**0.78 (0.77, 0.80)**	**<.01**	**0.95 (0.94, 0.96)**	**<.01** **	**18.01%**
Black segregation	**0.77 (0.76, 0.77)**	**<.01**	**0.78 (0.77, 0.80)**	**<.01**	**0.98 (0.97, 0.99)**	**<.01** **	**8.70%**
Hispanic segregation	**0.77 (0.76, 0.78)**	**<.01**	**0.80 (0.78, 0.81)**	**<.01**	**0.97 (0.95, 0.98)**	**<.01** **	**12.89%**
AI/AN segregation	**0.76 (0.75, 0.77)**	**<.01**	**0.78 (0.77, 0.79)**	**<.01**	**0.97 (0.96, 0.98)**	**<.01** **	**10.88%**
NH/OPI vs. white all‐cause mortality difference							
Neighborhood Deprivation	**0.96 (0.93, 0.98)**	**.01**	0.98 (0.95, 1.00)	.06	**0.98 (0.97, 0.99)**	**.01** **	**41.07%**
Black segregation	**0.96 (0.93, 0.98)**	**<.01**	**0.97 (0.94, 0.99)**	**.01**	**0.99 (0.98, 1.00)**	**.01** **	**27.08%**
Hispanic segregation	**0.96 (0.94, 0.98)**	**<.001**	0.98 (0.96, 1.01)	.6	**0.98 (0.96, 0.99)**	**<.01** **	**58.73%**
AI/AN segregation	**0.96 (0.93, 0.98)**	**<.01**	**0.97 (0.94, 0.99)**	**.01**	**0.99 (0.98, 1.00)**	**.02** **	**24.74%**
AI/AN vs. white all‐cause mortality difference: Mediation by black segregation, stratified by rurality							
Black segregation: Rural strata	**1.10 (1.05 ‐ 1.15)**	**<.001**	**1.08 (1.02 ‐ 1.14)**	**.01**	1.02 (1.00 ‐ 1.05)	.09*	22.35%
Black segregation: Urban strata	**1.05 (1.00 ‐ 1.09)**	**.05**	1.02 (0.96 ‐ 1.08)	.50	1.03 (0.99 −1.07)	.12	57.57%

Note

IOW denotes inverse odds weighting approach. Bold text denotes statistical significance at *P* < .05.

* indicates statistically significant indirect effect at *P* < .1

** indicates statistically significant indirect effects at *P* < .05. Models estimate with 500 bootstrap replications and adjusted for age, sex, individual SES, rurality, and medical and mental health comorbidities. Percent change was calculated as (total effect – direct effect)/total effect

Neighborhood deprivation, black segregation, Hispanic segregation, and AI/AN segregation all mediated mortality differences between Asians, Hispanics, and NH/OPIs compared to whites. Only black segregation mediated black‐white mortality differences.

### 3.3 Moderation

Black segregation significantly modified the AI/AN mortality disparity (interaction HR = 0.792, 95%CI: 0.628‐0.997) (Table 4). When stratified by race/ethnicity, as black segregation increased, mortality decreased among AI/ANs but increased among whites. AI/AN mortality decreased for those living in more black‐segregated neighborhoods, while mortality increased for whites with greater non‐Hispanic black segregation. There was no evidence of moderation by neighborhood deprivation (interaction HR = 1.002, 95%CI: 0.999‐1.005), Hispanic segregation (interaction HR = 0.994, 95%CI: 0.794‐1.245), and AI/AN segregation (interaction HR = 1.26, 95%CI: 0.93‐1.71).

**TABLE 4:** Modifying effects of neighborhood characteristics on racial/ethnic mortality differences relative to whites

	All‐cause mortality HR (95% CI)
Neighborhood deprivation	
Race/ethnicity	
AI/AN	0.84 (0.61‐1.15)
Asian	0.88 (0.67‐1.17)
Black	**0.75 (0.69**‐**0.82)**
vHispanic	1.03 (0.83‐1.27)
NH/OPI	0.80 (0.62‐1.04)
Neighborhood deprivation	**1.003 (1.003**‐**1.004)**
Race/ethnicity*neighborhood deprivation interaction	
AI/AN*deprivation	1.00 (0.999‐1.01)
Asian*deprivation	**0.997 (0.994**‐**0.9997)**
Black*deprivation	**1.002 (1.001**‐**1.003)**
Hispanic*deprivation	**0.997 (0.994**‐**0.9997)**
NH/OPI*deprivation	1.002 (0.999‐1.004)
Black segregation	
Race/ethnicity	
AI/AN	**1.12 (1.05**‐**1.19)**
Asian	**0.71 (0.61**‐**0.82)**
Black	**0.90 (0.88**‐**0.93)**
Hispanic	**0.76 (0.67**‐**0.87)**
NH/OPI	0.96 (0.89‐1.03)
Black segregation	**1.12 (1.07**‐**1.18)**
Race/ethnicity*Black interaction	
AI/AN*segregation	**0.79 (0.63**‐**0.997)**
Asian*segregation	0.71 (0.48‐1.05)
Black*segregation	1.01 (0.95‐1.08)
Hispanic*segregation	1.03 (0.74‐1.44)
NH/OPI*segregation	1.00 (0.84‐1.18)
Hispanic segregation	
Race/ethnicity	
AI/AN	**1.07 (1.02**‐**1.13)**
Asian	**0.72 (0.63**‐**0.82)**
Black	**0.94 (0.92**‐**0.96)**
Hispanic	**0.86 (0.80**‐**0.94)**
NH/OPI	**1.05 (1.01**‐**1.10)**
Hispanic segregation	0.98 (0.93‐1.04)
Race/ethnicity*Hispanic segregation	
AI/AN*segregation	0.99 (0.79‐1.25)
Asian*segregation	0.75 (0.51‐1.12)
Black*segregation	0.97 (0.91‐1.03)
Hispanic*segregation	**0.80 (0.65**‐**0.99)**
NH/OPI*segregation	**0.66 (0.56**‐**0.79)**
AI/AN segregation	
Race/ethnicity	
AI/AN	**1.05 (1.01, 1.10)**
Asian	**0.66 (0.59, 0.73)**
Black	**0.93 (0.91, 0.94)**
Hispanic	**0.75 (0.68, 0.83)**
NH/OPI	0.96 (0.92, 1.01)
AI/AN segregation	0.97 (0.87, 1.07)
Race/ethnicity*AI/AN segregation	
AI/AN*segregation	1.26 (0.93, 1.71)
Asian*segregation	0.91 (0.23, 3.5)
Black*segregation	1.10 (0.8, 1.40)
Hispanic*segregation	1.33 (0.92, 1.93)
NH/OPI*segregation	0.78 (0.43, 1.40)
Black segregation stratified by rurality	
Rural	
Race/ethnicity	
AI/AN	**1.15 (1.08**‐**1.22)**
Black segregation	**1.22 (1.15**‐**1.30)**
Race/ethnicity*Black interaction	
AI/AN*segregation	**0.72 (0.54**‐**0.97)**
Urban	
Race/ethnicity	
AI/AN	1.09 (1.00‐1.20)
Black segregation	**1.09 (1.03**‐**1.14)**
Race/ethnicity*Black interaction	
AI/AN*segregation	0.83 (0.60‐1.15)

Notes

Bold text indicates statistical significance at *P* < .05. Models controlled for age, sex, individual SES, rurality, and medical and mental health comorbidities

For the racial/ethnic groups with mortality advantages relative to whites, we found that associations between race/ethnicity and mortality varied by neighborhood characteristics. As neighborhood deprivation increased, mortality advantages increased for Asian and Hispanics, but were attenuated for blacks. The positive association between mortality and neighborhood deprivation was stronger among blacks than whites (interaction HR = 1.002, 95%CI: 1.001‐1.003). In more Hispanic‐segregated neighborhoods, mortality advantages were accentuated among Hispanics (interaction HR = 0.803, 95%CI: 0.654‐0.987) and NH/OPIs (interaction HR = 0.662, 95%CI: 0.558‐0.786).

### 3.4 Sensitivity analyses

Stratifying the black segregation mediation analysis by rurality, revealed that black segregation trended toward mediating AI/AN mortality disparities among rural Veterans (indirect effect HR = 1.02, 95%CI: 1.00‐1.05, *P*‐value = .09), but not among urban Veterans (indirect effect HR = 1.03, 95%CI: 0.99‐1.07, *P*‐value = .12) (Table 3).

In the urban‐rural stratified black segregation effect modification analysis, black segregation significantly modified AI/AN disparities only within the rural‐dwelling subpopulation, in a similar direction and magnitude as in the entire sample (interaction HR = 0.72, 95%CI: 0.54‐0.97) (Table 4).

In CHSDA counties, similar to findings in the main analysis, AI/AN segregation did not moderate AI/AN disparities (interaction HR = 1.28, 95%CI: 0.95‐1.73), but did mediate AI/AN disparities (indirect effect HR = 1.03, 95%CI: 1.00‐1.06, *P*‐value = .02) (Tables S2‐S3).

### 3.5 Stratified mediation analyses

Based on our initial findings that black segregation mediated AI/AN disparities and attenuated AI/AN disparity in more black‐segregated communities, we conducted supplementary mediation analysis of black segregation, stratified by high (>33 percent black isolation) and low (≤33 percent black isolation) black segregation counties. We determined high and low segregation based on the level of black isolation where AI/AN and white mortality hazard ratios were equivalent. We found that black segregation mediated AI/AN disparities among Veterans living in counties with low black segregation (indirect effect HR = 1.04, 95%CI:1.01‐1.06, *P*‐value < .01), but not in high black‐segregated counties (Table S4).

## 4 DISCUSSION

We built upon prior work on racial/ethnic mortality disparities among VHA‐users^2^, ^4^ to characterize racial/ethnic mortality differences and then explore the role of two neighborhood characteristics—SES and segregation—in explaining residual disparities within this population with access to health care. Overall, we found all‐cause mortality disparities among AI/ANs only, relative to whites, after accounting for individual‐level social determinants and health differences, whereas we found mortality advantages for most other racial/ethnic minority groups. This AI/AN disparity appeared to be both partially mediated and moderated by black segregation and mediated by AI/AN segregation, but not by neighborhood deprivation or Hispanic segregation.

Studies among VHA‐users have identified some conditions and disease area where blacks experience similar or better mortality outcomes than whites.^2^, ^4^, ^7^, ^8^ However, we found that black VHA‐users still experienced higher mortality after accounting for age and sex differences. However, with further adjustment for individual SES and rurality, blacks had a mortality advantage relative to whites. Individual SES and rurality are both important social determinants related to mortality.^47^, ^48^ That black mortality disparities no longer existed *after* we accounted for these social determinants suggests that they may contribute to black‐white mortality differences within this population with health care access.

There is a continual and pressing need to address AI/AN mortality disparities, which are also well‐documented in the US general population, as this population has endured a legacy of historic conflict, trauma, and injustice.^24^ European and American colonizers forcibly removed AI/ANs from their native lands and deliberately sought to destroy their cultural practices, spiritual beliefs, and family systems.^23^, ^24^ The effects of these racist policies and practices endure today, resulting in high levels of discrimination, poverty and emotional and physical trauma among AI/ANs,^23^ and AI/AN reservations located in rural areas with few economic opportunities and resources.^23^ In this sample with health care access, AI/AN‐white mortality disparities existed, even after accounting for individual SES, rurality, and medical and mental health differences, which prompted us to explore the role of neighborhood‐level factors. To our knowledge, this study is the first to examine neighborhood‐level mediators of AI/AN all‐cause mortality disparities.

We found that black residential segregation both moderated and partially mediated AI/AN VHA‐user mortality disparities. Subsequent stratified mediation analyses uncovered that black segregation partially mediated AI/AN disparities in low black‐segregated communities, but not in high black‐segregated communities. In fact, in high black‐segregated communities, AI/AN mortality disparities may be attenuated. For example, in the Stroke Belt, a southeast region in United States with high black segregation,^49^ AI/AN age‐ and sex‐standardized mortality rates are similar to whites, whereas AI/AN mortality rates are significantly higher than whites outside of the Stroke Belt, where black segregation is lower (Table S5). These AI/AN‐white mortality differences may also exist in other high and low black‐segregated communities. In less black‐segregated communities, black segregation may be a proxy for other related neighborhood characteristics, such as health care access and quality, crime, and social cohesion, that may account for AI/AN disparities. To our knowledge, there is minimal research on the experiences of AI/ANs living in black communities, including for experiences of acculturation, discrimination, or buffering against racism, and limited research on whether living in these communities might be protective of AI/AN mortality. While neighborhood‐based interventions can target high black‐segregated neighborhoods to improve health among all residents, they should also ensure that they do not inadvertently create disparities, if for example, whites benefit more from these efforts than AI/ANs. Neighborhood‐based interventions in less black‐segregated communities can focus more specifically on AI/ANs.

We also found that black segregation's mediation and moderation effects on AI/AN disparities were also limited to rural areas. AI/ANs are more likely to reside in rural areas than other racial/ethnic groups. Our finding that neighborhood factors contributing to AI/AN disparities differ by rurality support existing research that urban AI/AN experiences are different from their rural counterparts, such as greater challenges in accessing culturally competent care, loss of cultural ties, historical trauma from forced integration, and higher rates of depression and metabolic conditions.^50^ Efforts to address neighborhood‐level social determinants for AI/ANs of health should be tailored by rurality. Black segregation's mediation effects in rural areas is consistent with our finding of mediation in less black‐segregated areas as rural areas often also have lower levels of black segregation compared to urban areas.

We also found that AI/AN segregation mediated AI/AN mortality among residents of CHSDA county, who reside on or near reservations.^24^ Prior research has identified both beneficial and detrimental aspects of living on reservations: while reservations are often located in rural areas with high poverty and unemployment,^24^ living on reservations allows individuals to maintain tribal and cultural ties.^25^ While we could not determine whether AI/AN VHA‐users lived on reservations, it is possible that within CHSDA counties, those in lower AI/AN segregation communities resided *near* but not on reservations, which allowed them to maintain cultural ties, while taking advantage of economic opportunities off reservations. It may also be easier for these individuals living off reservations to access VHA care.^51^

Among other racial/ethnic groups with mortality advantages relative to whites, neighborhood characteristics mediated and moderated some of these relationships. Mediating effects were relatively consistent across racial/ethnic groups: neighborhood racial/ethnic segregation and SES partially mediated mortality advantages for Asians, Hispanics, and NH/OPIs versus whites. However, only black segregation mediated blacks’ mortality advantages.^52^ We found greater racial/ethnic variation in the neighborhood characteristics’ modifying effects. Living in lower SES neighborhoods was protective of mortality among Hispanics and Asians but deleterious for blacks, relative to whites. Living in Hispanic‐segregated neighborhoods conferred mortality protection for NH/OPIs and Hispanics. The latter relationship supports the Hispanic ethnic density effect: Hispanics living in neighborhoods with a higher Hispanic concentration are healthier.^53^, ^54^ These findings provide additional evidence of neighborhoods being an important social determinant of health and disparities, but these relationships are complex and vary by racial/ethnic group.^55^, ^56^ Developing neighborhood‐level policies to improve health may require nuanced strategies tailored to different racial/ethnic groups.

Our study had several limitations. We used Veteran addresses from a single time point at the start of the observation period and were unable to account for those who subsequently moved. However, research has demonstrated consistency over time in neighborhood characteristics for older individuals.^57^ Our neighborhood data for both ADI and segregation predated our mortality ascertainment. While use of this data ensured that neighborhood exposure preceded mortality outcomes, it is possible that neighborhoods may have changed between when neighborhood exposure data were ascertained and this study's observation period. Race/ethnicity misclassification could have occurred in the CDC mortality files, especially for AI/ANs.^58^ For Asian and Hispanic subgroups, we could not control for English proficiency or national origin. Differences in individual (Table S6) and neighborhood‐level characteristics between those included and excluded due to missing census tract identifiers (from PO Box, island residence, missing and rural route addresses) might lead to selection bias, but AI/AN mortality disparities were similar in included and excluded samples (Table S7). VHA‐users differ from the US general population on both health facilitating and health limiting characteristics. VHA‐users access VHA health care, have lower SES and poorer health, and are older and more likely to be male. Access to health care in our sample may mitigate neighborhood influences on health, but lower SES and poorer health may make them more susceptible to detrimental effects of neighborhood social determinants of health. There may be selection bias of who chooses to serve in the military and/or use VHA care.

This study among VHA‐users provides a unique opportunity to consider how health care access may mitigate disparities, and how factors beyond health care contribute to these disparities. Our results add to a growing body of evidence that closing disparity gaps will require more than improvements in health care and health care access, to also address social determinants of health. Health care systems are part of a broader network of factors, including social services that can address these determinants. While the VHA can continue targeted approaches to improve AI/AN care, such as ensuring culturally appropriate care and tailoring behavioral interventions (eg, alcohol cessation programs), it can also consider creative ways to look beyond health care to impact health outcomes.

Future research can explore the experience of AI/ANs residing in black‐segregated communities, including their experiences of acculturation and discrimination, underlying mechanisms through which black segregation is related to AI/AN health disparities, especially in high black‐segregated communities, and other neighborhood‐level social determinants of health that may contribute to urban AI/AN disparities. Research on social determinants influencing AI/AN disparities should consider urban‐rural differences. These efforts can inform future social determinant‐oriented interventions, such as improving AI/AN social support from other Veterans (eg, Veteran service organizations or AI/AN Veteran groups), and ways that health systems might partner with community‐based organizations.

Health care systems, including VHA, are the primary entity charged with improving health of populations and increasingly held accountable to address health disparities. Closing this gap may require health care systems to explore novel collaborations with social services to address neighborhood‐level social determinants of health, such as community partnerships, financing strategies that reimburse social services, and co‐locating resources (eg, farmers markets or parks) in or near health care settings.

## CONFLICT OF INTEREST

The authors declare no conflicts of interest.

## Supporting information

Supplementary Material

Table S1‐S7
