# Supplementary material for: Relationship of neighborhood social determinants of health on racial/ethnic mortality disparities in US veterans—Mediation and moderating effects
Source: Health Serv Res. 2020 Aug 29;55(Suppl 2):851–62. doi: 10.1111/1475-6773.13547 (PMC7518818; doi:10.1111/1475-6773.13547)
Supplement: Supplementary file 2 — Table S1‐S7 [file HESR-55-851-s002.docx]

**Table S1. Adjusted Cox regression results examining racial/ethnic differences in all-cause mortality**

|  | Model 1  HR (95% CI) | Model 2  HR (95%CI) | Model 3 HR (95% CI) |
| --- | --- | --- | --- |
| Race/ethnicity | | | |
| Non-Hispanic White | Ref | Ref | Ref |
| AI/AN | **1.15 (1.10, 1.19)** | **1.10 (1.105, 1.14)** | **1.07 (1.03 – 1.11)** |
| Asian | **0.63 (0.58, 0.68)** | **0.61 (0.55, 0.68)** | **0.67 (0.60 – 0.74)** |
| Non-Hispanic Black | **1.02 (1.00, 1.04)** | **0.96 (0.94., 0.97)** | **0.93 (0.91 – 0.94)** |
| Hispanic | **0.81 (0.76, 0.86)** | **0.74 (0.68, 0.81)** | **0.75 (0.68 – 0.82)** |
| NH/OPI | 0.98 (0.92, 1.04) | 0.97 (0.93, 1.02) | **0.95 (0.91 – 0.99)** |
| Age Category | | | |
| < 30 | Ref | Ref | Ref |
| 30 – 39 | **1.55 (1.47, 1.63)** | **1.59 (1.51, 1.68)** | **1.52 (1.45 – 1.60)** |
| 40 – 49 | **3.95 (3.76, 4.15)** | **4.06 (3.86, 4.26)** | **3.56 (3.40 – 3.74)** |
| 50 – 59 | **10.45 (0.98, 10.95)** | **10.74 (10.27, 11.24)** | **8.46 (8.09 – 8.84)** |
| 60 – 69 | **14.56 (13.88, 15.28)** | **15.33 (14.62, 16.07)** | **12.52 (11.95 – 13.12)** |
| 70 – 79 | **31.89 (30.35, 33.51)** | **35.40 (33.75, 37.12)** | **30.73 (29.29 – 32.24)** |
| 80+ | **74.83 (71.01, 78.85)** | **81.74 (77.75, 85.95)** | **74.67 (70.98 – 78.56)** |
| Sex | | | |
| Male | Ref | Ref | Ref |
| Female | **0.67 (0.65, 0.68)** | **0.66 (0.65, 0.68)** | **0.70 (0.69 – 0.72)** |
| Individual SES | | | |
| High SES |  | Ref | Ref |
| Low SES |  | **1.48 (1.46, 1.50)** | **1.32 (1.30 – 1.34)** |
| Unknown |  | **1.34 (1.32, 1.36)** | **1.22 (1.21 – 1.24)** |
| Rurality indicator | | | |
| Urban |  | Ref | Ref |
| Rural |  | **0.98 (0.96, 0.99)** | **0.97 (0.96 – 0.98)** |
| Highly rural |  | **0.95 (0.93, 0.98)** | **0.96 (0.93 – 1.00)** |
| Mental health comorbidity | | | |
| No mental health diagnosis |  |  | Ref |
| SMI |  |  | **1.51 (1.49 – 1.54)** |
| Depression without SMI |  |  | **1.15 (1.14 – 1.16)** |
| Other mental health diagnosis |  |  | **1.17 (1.15 – 1.19)** |
| Medical comorbidity |  |  | **1.16 (1.16 – 1.17)** |

Notes: Bold text denotes statistically significant association at p<0.05. Model 1 covariates included race/ethnicity, age and sex. Model 2 covariates included race/ethnicity, age, sex, individual SES, and rurality. Model 3 covariates included race/ethnicity, age, sex, Individual SES, rurality, and medical and mental health comorbidities.

**Table S2. AI/AN segregation effect modification effects on racial/ethnic differences in mortality among VHA-users residing in CHSDA counties**

|  | Hazard ratio (95%CI) |
| --- | --- |
| Race/ethnicity | |
| AI/AN | 1.09 (1.02, 1.16) |
| Asian | 0.58 (0.48, 0.70) |
| Black | 0.91 (0.89, 0.94) |
| Hispanic | 0.80 (0.76, 0.84) |
| NH/OPI | 0.94 (0.89, 0.99) |
| AI/AN segregation | 0.98 (0.87, 1.09) |
| Race/ethnicity * AI/AN segregation | |
| AI/AN * segregation | 1.28 (0.95, 1.73) |
| Asian * segregation | 2.03 (0.75, 5.53) |
| Black * segregation | 1.18 (0.88, 1.57) |
| Hispanic * segregation | 1.03 (0.86, 1.22) |
| NH/OPI * segregation | 0.84 (0.45, 1.58) |

Notes: CHSDA Counties denote the Indian Health Service’s Contract Health Service Delivery Area. Bold text indicates statistical significance at p<0.05. Models controlled for age, sex, individual SES, rurality, and medical and mental health comorbidities

**Table S3. AI/AN segregation mediating effects on AI/AN vs. White mortality differences among VHA-users residing in CHSDA counties**

|  | Total Effect | | Direct effect | | Indirect effect | | % change |
| --- | --- | --- | --- | --- | --- | --- | --- |
|  | HR (95% CI) | p-value | HR (95% CI) | p-value | HR (95% CI) | P-value |  |
| *AI/AN vs. White all-cause mortality differences* | | | | | | | |
| AI/AN segregation | **1.12**  **(1.07, 1.18)** | **<0.05** | **1.09**  **(1.03, 1.14)** | **<0.05** | **1.03**  **(1.00, 1.06)** | **0.02** | 27.2% |

Notes: Bold text indicates statistical significance at p<0.05. Model controlled for age, sex, individual SES, rurality, and medical and mental health comorbidities.

**Table S4: Black segregation IOW mediation effects on AI/AN vs. White mortality differences among VHA-users, stratified by participants living in high (>33% Black isolation) and low (≤33% Black isolation) Black-segregated counties**

|  | Total Effect | | Direct effect | | Indirect effect | | % change |
| --- | --- | --- | --- | --- | --- | --- | --- |
|  | HR (95% CI) | p-value | HR (95% CI) | p-value | HR (95% CI) | P-value |  |
| *AI/AN vs. White all-cause mortality differences* | | | | | | | |
| High Black segregation | 1.02  (0.94, 1.09) | 0.67 | 1.02  (0.92, 1.12) | 0.74 | 1.00  (0.94, 1.06) | 0.98 | -5.08% |
| Low Black segregation | **1.09**  **(1.05, 1.13)** | **<0.01** | **1.05**  **(1.01, 1.10)** | **0.02** | **1.04**  **(1.01, 1.06)** | **0.01** | 40.67% |

Notes: Bold text indicates statistical significance at p<0.05. Model controlled for age, sex, individual SES, rurality, and medical and mental health comorbidities. For the high Black segregation strata, we combined highly rural and rural to create a 2-category measure of rurality: urban vs. rural/ highly rural due to a small sample in the highly rural category.

**Table S5: AI/AN and White VHA-user age- and sex-standardized mortality in Stroke Belt states (high Black segregation) and non-Stroke Belt states (low Black segregation)**

|  | **Black isolation** | **Standardized mortality rate^1^** | **Standardized mortality ratio** |
| --- | --- | --- | --- |
| *Stroke Belt States* | | | |
| AI/AN | 0.38 | 5115.89 | 0.98 (0.87, 1.10) |
| White |  | 5196.20 | 1.00 (ref) |
| *Non-Stroke Belt States* | | | |
| AI/AN | 0.22 | 5290.04 | 1.09 (1.05, 1.13) |
| White |  | 4860.46 | 1.00 (ref) |

Note: 1. Standardized mortality rates standardized to the fiscal year 2009 non-Hispanic White VHA-user population; 2. Standardized mortality ratios calculated as the ratio of the AI/AN to White (reference) standardized mortality rates; 3. Stroke Belt states include the following 8 states in the Southeast United States: Alabama, Arkansas, Georgia, Louisiana, Mississippi, North Carolina, South Carolina, and Tennessee, and non-Stroke Belt states include the rest of the United States

**Table S6:** **Comparison of characteristics of individuals included in sample and excluded due to missing or non-matching census tract identifiers**

|  | Included in sample | Excluded due to missing/non-matching census tract identifier  (n=568,967) | p-value |
| --- | --- | --- | --- |
| Race/ethnicity, % | | | |
| AI/AN | 0.38 | 1.15 | <0.001 |
| Asian | 0.66 | 0.78 |  |
| Black | 14.56 | 12.15 |  |
| Hispanic | 4.42 | 7.93 |  |
| NH/OPI | 0.55 | 0.80 |  |
| White | 75.15 | 72.68 |  |
| Multi-race/unknown | 4.29 | 4.51 |  |
| Individual SES, % | | | |
| High | 25.64 | 21.77 | <0.001 |
| Low | 27.05 | 30.90 |  |
| Indeterminate | 47.32 | 47.33 |  |

Note: Source of missing census tract identifiers: PO Box/rural route/highway contract address (79%, n = 488,410); island residence (5%, n=29,593); missing address (5%, n=27,444); other (12%, n=65,787)

**Table S7: Comparison of AI/AN vs. White all-cause mortality disparities between individuals included in the analysis and those excluded due to missing census tract identifiers**

|  | HR (95% CI) |
| --- | --- |
| **Model 1** | |
| AI/AN | **1.16 (1.11 – 1.21)** |
| Missing | **1.08 (1.06 – 1.09)** |
| AI/AN * Missing | 0.93 (0.95 – 1.02) |
| **Model 2** | |
| AI/AN | **1.10 (1.06 – 1.15)** |
| Missing | **1.06 (1.05 – 1.08)** |
| AI/AN * Missing | 0.95 (0.86 – 1.04) |
| **Model 3** | |
| AI/AN | **1.07 (1.03 – 1.12)** |
| Missing | **1.05 (1.04 – 1.07)** |
| AI/AN * Missing | 0.99 (0.92 – 1.06) |

Note: Bold text denotes statistically significant association at p<0.05. Model 1 adjusted for age and sex, Model 2 adjusted for age, sex, individual SES, and rurality. Model 3 adjusted for age, sex, Individual SES, rurality, and medical and mental health comorbidities.
